# Periodic CO_2_ Dosing Strategy for *Dunaliella salina* Batch Culture

**DOI:** 10.3390/ijms160511509

**Published:** 2015-05-19

**Authors:** Kezhen Ying, D. James Gilmour, William B. Zimmerman

**Affiliations:** 1 Department of Chemical and Biological Engineering, University of Sheffield, Mappin Street, Sheffield S1 3JD, UK; E-Mail: w.zimmerman@sheffield.ac.uk; 2 Department of Molecular Biology and Biotechnology, University of Sheffield, Firth Court, Western Bank, Sheffield S10 2TN, UK; E-Mail: d.j.gilmour@sheffield.ac.uk

**Keywords:** periodic CO_2_ dosing, *D. salina*, dosing time, dosing interval, CO_2_ capture efficiency

## Abstract

A periodic CO_2_ dosing strategy for *D. salina* 19/30 batch culture is proposed. A model of periodic CO_2_ dosing including dosing time calculation, dosing interval estimation and final chlorophyll yield prediction was established. In experiments, 5% CO_2_/95% N_2_ gas was periodically dosed into *D. salina* culture. Two different gas dosing flow rates were tested. The corresponding dosing time for each flow rate was estimated via the model (10 min·d^−1^ for 0.7 L·min^−1^ and 36 min·d^−1^ for 0.3 L·min^−1^). Daily pH measurements showed that the pH of these cultures dosed periodically was always kept between 7.5 and 9.5, which highlights that periodic gas supply can maintain a suitable range of pH for microalgal growth without expensive buffers. Notably the culture dosed for set daily intervals was seen to have similar growth to the culture supplied constantly, but with much higher CO_2_ capture efficiency (11%–18%) compared to continuous dosing (0.25%). It shows great potential for using periodic gas supply to reduce cost, wasted gas and energy use.

## 1. Introduction

Currently, one emerging application of microalgae is for the fixation of CO_2_ [1], for it may offer a way to reduce the levels of unwanted CO_2_ whilst also allowing the production of useful by-products from the algae such as bio-oils, chemicals, fatty acids and substances, *etc.* [2]. However, the large-scale cultivation of microalgae still faces many problems and barriers. These include the energy required to continuous mix, dewater and process the algal biomass, and the large areas of land needed for such projects [3]. CO_2_ dosing is one of the concerns highlighted in this study. The major problem with CO_2_ being dosed into microalgae cultures is that conventionally the bubbles used are fairly large. As Zimmerman *et al.* [4] discuss, such bubbles will reduce the interfacial area between bubbles and the liquid, and will reduce the overall mass transfer for both CO_2_ dissolution and O_2_ stripping. To counteract the resulting low mass transfer, large flow rates of CO_2_ enriched gas are often continuously bubbled into the cultures. While the mixing effect on the culture is beneficial for ensuring the even distribution of light and nutrients, the intense turbulence produced by large flow rate can damage the algal cells within the culture and reduce the productivity [5]. A further disadvantage of using high flow rate, especially with low mass transfer, is that most of the gas bubbled into the culture will pass through and be wasted. Carvalho and Malcata [6] agree that when CO_2_ is bubbled into algal cultures, the mass transfer is not particularly effective and considerable gas is wasted, adding to operational costs. Even with some high mass transfer dosing techniques (e.g., Dissolved Air Flotation systems), hypothetically, continuous dosing is still not a wise option, because when the concentration of dissolved CO_2_ has reached an equilibrium value, the gas-liquid mass transfer process stops. Any additional input beyond this point would not increase the total amount of dissolved CO_2_, but cost energy and waste CO_2_.

An innovative CO_2_ microbubble-dosing technology was studied by Ying *et al.* [7,8,9], which has proved that the microbubble-dosing technology can enable a higher CO_2_ mass transfer and consequently lead to a higher microalgal growth rate and a greater CO_2_ capture efficiency. Nonetheless, the operational parameters (e.g., dosing time, dosing interval and flow rate, *etc.*) for microbubble-dosing technology still need to be engineered. In this study, an optimal periodic CO_2_ dosing strategy is proposed and a model established based on *D. salina* (19/30) cultures. The main hypothesis for this study is that by using a microbubble driven airlift bioreactor, high mass transfer can be attained in a batch culture by supplying gas periodically (with little CO_2_ wasted and less energy cost) and achieve similar algal growth compared to when the gas is supplied continuously.

## 2. Results and Discussion

### 2.1. Model of Periodical CO_2_ Dosing

For an optimal periodical dosing strategy, three major principles need to be followed. Firstly, despite the exclusion of buffer solution and pH auto-regulating system, the pH of the culture needs to be controlled in a suitable range by periodic CO_2_ dosing. Secondly, the dosing time should only be long enough for CO_2_ to reach its equilibrium concentration. Meanwhile, the equilibrium pH (corresponding to CO_2_ equilibrium concentration) is expected to be the lower limit of the suitable pH range for the microalgal species being utilized. Thirdly, the time period without dosing (dosing interval) should ensure that the microalgae use up the dosed CO_2_, whilst ensuring that the pH increase to the upper limit is within the suitable pH range. Following these three principles, a culture with periodic dosing, compared to one with continuous dosing, should have sufficient (but not excess) CO_2_ and an optimal pH range to support optimal growth (achieving similar growth as with continuous dosing), while with minimal amount of CO_2_ wasted and less energy input. Based on these three principles, the dosing time, dosing interval and final algal yield can be estimated.

#### 2.1.1. Estimation of Dosing Time

Assuming the suitable pH range for the culture of a particular microalga species is given between A and B, of which the corresponding concentration of [CO_2_] can be calculated by Equation (1) [7,8,9,10,11].

(1) $$[{\text{CO}}_{\text{2}}]=\frac{(10^{\text{-pH}}-10^{\text{(pH-14)}}+\Delta [{\text{Na}}^{+}])10^{\left(-2\text{pH}\right)}}{10^{\left(-6.381-\text{pH}\right)}+2\times 10^{\left(-16.758\right)}}\begin{array}{c} \left(\text{mol/L}\right) \end{array}$$

To control the culture in a target pH range the amount of CO_2_ needed to be transferred to the medium can simply be estimated as the difference between dissolved CO_2_ level at pH=B and pH=A (assuming the CO_2_ uptake rate is negligible compared with CO_2_ gas-liquid mass transfer rate). The dosing time is thereby calculated as the amount of CO_2_ needed to be transferred to the medium divided by the average CO_2_ mass transfer rate, shown in Equation (2), where ν'_MTR_ represents CO_2_ average mass transfer rate (mol·L^−1^·min^−1^) and can be calculated as Equation (3). The derivation of Equation (3) was explained in Ying *et al.* [9]. Finally, assuming the pH before dosing was controlled at B which gives the initial dissolved CO_2_ level as [CO_2_]_pH=B_, the optimal valid dosing time needed to drop the pH from B to A can then be estimated through Equation (4), which is obtained by combining Equations (2) and (3). As long as the suitable pH range for a particular type of algae is given and the K_L_a for a certain dosing condition is known, the optimal dosing time can be estimated.

(2) $$t_{d}=\frac{{\left[{\text{CO}}_{2}\right]}_{\text{pH=A}}-{\left[{\text{CO}}_{2}\right]}_{\text{pH=B}}}{{\nu \prime }_{\text{MTR}}}$$

(3) $${\text{v′}}_{\text{MTR}}=\frac{{\text{K}}_{\text{L}}\text{a}({\left[{\text{CO}}_{2}\right]}^{*}-{\left[{\text{CO}}_{2}\right]}_{0})}{\frac{{\text{K}}_{\text{L}}\text{a}t_{\text{d}}}{2}+1}$$

(4) $$t_{\text{d}}=\frac{{\left[{\text{CO}}_{2}\right]}_{\text{pH=A}}-{\left[{\text{CO}}_{2}\right]}_{\text{pH=B}}}{{\text{K}}_{\text{L}}\text{a}({\left[{\text{CO}}_{2}\right]}^{*}-{\left[{\text{CO}}_{2}\right]}_{\text{pH=B}})-\frac{1}{2}{\text{K}}_{\text{L}}\text{a}({\left[{\text{CO}}_{2}\right]}_{\text{A}}-{\left[{\text{CO}}_{2}\right]}_{\text{pH=B}})}$$

From the previous study carried out by Ying *et al.* [9], it was found that K_L_a estimation is more accurate based on the changes in [*C*_T_], especially when pH > 8.4. Therefore, Equation (4) can also be written in terms of [*C*_T_], shown as Equation (5).

(5) $$t_{\text{d}}=\frac{{\left[C_{\text{T}}\right]}_{\text{pH=A}}-{\left[C_{\text{T}}\right]}_{\text{pH=B}}}{{\text{K}}_{\text{L}}\text{a}({\left[C_{\text{T}}\right]}^{*}-{\left[C_{\text{T}}\right]}_{\text{pH=B}})-\frac{1}{2}{\text{K}}_{\text{L}}\text{a}({\left[C_{\text{T}}\right]}_{\text{A}}-{\left[C_{\text{T}}\right]}_{\text{pH=B}})}$$

#### 2.1.2. Estimation of Dosing Interval

The dosing interval here is defined as the time period without gas dosing. During this period, pH increases gradually because of the uptake of CO_2_ by microalgae, until the pH achieves the upper limit of the suitable range, then dosing needs to be started again. Therefore, the effective estimation of dosing interval is crucial for periodic dosing, either too long or too short could cause the pH to exceed the upper or lower limit of the suitable range and adversely affect the algal growth.

The simplest way to estimate the dosing interval is to divide the amount of CO_2_ expected to be absorbed by the CO_2_ uptake rate. However, the instantaneous growth rate differs with the concentration of the algae [12], which leads to changes in CO_2_ uptake rate. Instead of instantaneous CO_2_ uptake rate, the average CO_2_ uptake rate for the whole active growth period is therefore used to simplify the estimation of dosing interval Equation (6).

(6) $$t_{\text{i}}=\frac{{\left[{\text{CO}}_{2}\right]}_{\text{pH=A}}-{\left[{\text{CO}}_{2}\right]}_{\text{pH=B}}}{{\nu \prime }_{\text{CO2uptake}}}$$

Ying *et al.* [9] reported that when the pH was less than 8.4, the changes in the amount of total carbon almost all come from the changes in dissolved CO_2_ [13], however, when pH was more than 8.4, the changes in dissolved CO_2_ cannot fairly represent the CO_2_ uptake by algae, as both HCO_3_^−^ and CO_3_^2−^ would generate dissolved CO_2_ to compensate for the consumption of CO_2_. In other words, the amount of CO_2_ consumed by algae should be more than the changes in dissolved CO_2_, as HCO_3_^−^ and CO_3_^2−^ would also contribute to the amount of CO_2_ consumption. Therefore, the changes in total carbon [*C*_T_] should be considered instead of the changes in [CO_2_]. The [*C*_T_] can be calculated by Equation (7) [9]. Equation (6) should be converted into Equation (8). The dosing interval can then be estimated as long as the average CO_2_ uptake rate is known.

(7) $$\left[C_{\text{T}}]=[{\text{CO}}_{\text{2}}]+[{{\text{HCO}}_{3}}^{-}]+[{{\text{CO}}_{3}}^{2-}]=[{\text{CO}}_{2}]\cdot (1+10^{\text{pH}-\text{6.381}}+10^{2\text{pH}-\text{16.758}}\right)$$

(8) $$t_{\text{i}}=\frac{\Delta [C_{\text{T}}]}{{v\prime }_{\text{CO2uptake}}}=\frac{{\left[C_{\text{T}}\right]}_{\text{pH=A}}-{\left[C_{\text{T}}\right]}_{\text{pH=B}}}{{v\prime }_{\text{CO2uptake}}}$$

Since the periodic dosing strategy is proposed to achieve similar algal growth to when gas is supplied continuously, the average CO_2_ uptake rate is assumed to be the same as in the culture with continuous or excessive CO_2_ dosing. According to the information from previous *D. salina* cultures [7], the correlations for CO_2_ uptake rate *versus* algal biomass concentration (measured as chlorophyll content) was described by Equation (9) [7], and the relation between total chlorophyll content increase and total CO_2_ uptake was given (based on the cultures with excessive gas dosing) as Equation (10) [7].

(9) $${\text{v}}_{{\text{CO}}_{\text{2uptake}}}=7\times 10^{-5}\times [\text{Chl}]$$

(10) $$\Delta [\text{Chl}]=2703.4\times \Delta {\left[{\text{CO}}_{2}\right]}_{\text{uptak}e}=2703.4\times \Delta {\left[{\text{CO}}_{2}\right]}_{\text{dosed}}$$

For the same time period, Equation (10) can be transformed into Equation (11):

(11) $${\text{v′}}_{\text{Chl}}=2703.4\times {v\prime }_{{\text{CO}}_{\text{2uptake}}}$$

The average CO_2_ uptake rate can be fairly described as:

(12) $${v\prime }_{\text{CO2uptake}}=\int_{t_{\text{c1}}}^{t_{\text{c2}}}\frac{v_{\text{CO2uptake}}}{t_{\text{c}}}\text{d}t$$

where *t*_c_ is the selected culture time period (*t*_c2_–*t*_c1_), beginning from *t*_c1_ (the start of log growth phase) and ending by *t*_c2_ (the end of log growth phase).

Assuming the chlorophyll content ([Chl], mg·L^−1^) is equal to the initial concentration ([Chl]_0_, mg·L^−1^) plus the amount of its increase (ν'_Chl_ × *t*),

(13) $$[\text{Chl}]={\left[\text{Chl}\right]}_{0}+{v\prime }_{\text{Chl}}\cdot t$$

The average CO_2_ uptake rate can then be obtained by solving Equation (9), Equation (11)–(13), which gives:

(14) $${v\prime }_{\text{CO2uptake}}=\frac{7\times 10^{-5}\cdot {\left[\text{Chl}\right]}_{0}}{1-0.0946\cdot t_{\text{c}}}$$

The dosing interval is then given by:

(15) $$t_{\text{i}}=\frac{{\left[C_{\text{T}}\right]}_{\text{pH=A}}-{\left[C_{\text{T}}\right]}_{\text{pH=B}}}{7\times 10^{-5}\cdot {\left[\text{Chl}\right]}_{0}}\cdot (1-0.0946\cdot t_{\text{c}})$$

Theoretically the dosing interval is better to be shortened as the algae grows, which may in practice increase the complexity of the time control process. Using a constant dosing interval through the whole log growth period can simplify the operating process. By doing so, one of the major concerns is that the pH level may exceed the upper limit of a target range. However, one magnitude of difference in the concentration (mol·L^−1^) of dissolved CO_2_ only changes the pH by one unit [7], while the CO_2_ uptake rates for *D. salina* cultures are in the range of 10^−4^ to 10^−3^ mol·L^−1^·d^−1^, therefore, the pH value may only increase over the upper limit of a target range by no more than 0.1 unit. Plus empirically the target pH range for algal culture can be slightly narrowed down during the dosing interval calculation in order to prevent the pH value exceeding the real upper limit. The second concern is dissolved oxygen accumulation. According to the basic photosynthetic reaction equation, the O_2_ generation rate equals to CO_2_ uptake rate. Therefore, 10^−3^ mol·L^−1^·d^−1^ of CO_2_ uptake rate could result in around 32 mg·L^−1^ of daily O_2_ accumulation (300% over saturation with respect to air), which will diminish the rate of photosynthesis [14]. Thus, the dosing interval has to be limited to no more than 1 day so that Dissolved Oxygen (DO) can be removed by CO_2_ dosing in time. In terms of O_2_ stripping, it takes less than 10 min to reduce the dissolved oxygen from 32 to 0.03 mg L^−1^ by microbubble (d_32_ = 388 μm) dosing of 5% CO_2_/95% N_2_ under 0.7 L·min^−1^, according to the previous mass transfer study [7]. 

Statistically, from previous *D. salina* studies [7], the active growth phase usually lasted for 8 days (due to the light limitation). According to the literature, *D. salina* can tolerate a pH range of 5.5 to 10 [15]. A target pH range 7.5–9.5 was selected in this study. The dosing interval for *D. salina* cultures was estimated via Equation (15) to give approximately 1.5 days (based on Chl_0_ = 5 mg·L^−1^ and *t*_c_ = 8 days). Conservatively, the dosing interval of 1 day was suggested for best results in practice, so that DO can be removed by CO_2_ dosing in time.

#### 2.1.3. Prediction of Final Concentration of Chlorophyll Content 

Once the dosing time *t*_d_ and dosing interval *t*_i_ are known, the total dosing time (*t*_Total_) through the culture period *t*_c_ can be calculated as: *t*_d_ × *t*_c_/(*t*_d_ + *t*_i_). The total amount of CO_2_ uptake can be predicted by Equation (16):

(16) $$\Delta {\left[{\text{CO}}_{2}\right]}_{\text{uptake}(\text{Total})}={\text{v′}}_{\text{MTR}}\times t_{\text{Total}}=\frac{{\text{K}}_{\text{L}}\text{a}({\left[{\text{CO}}_{2}\right]}^{*}-{\left[{\text{CO}}_{2}\right]}_{0})}{\frac{{\text{K}}_{\text{L}}\text{a}\cdot t_{\text{d}}}{2}+1}\times \frac{t_{\text{d}}\cdot t_{\text{c}}}{t_{\text{d}}+t_{\text{i}}}$$

The final chlorophyll content can be estimated by Equation (17),

(17) $$\left[\text{Chl}]={\left[\text{Chl}\right]}_{0}+\Delta [\text{Chl}\right]$$

By combining Equation (11), Equations (16) and (17), it gives:

(18) $$[\text{Chl}]={\left[\text{Chl}\right]}_{0}+2703.4\times \frac{{\text{K}}_{\text{L}}\text{a}({\left[{\text{CO}}_{2}\right]}^{*}-{\left[{\text{CO}}_{2}\right]}_{0})}{\frac{{\text{K}}_{\text{L}}\text{a}\cdot t_{\text{d}}}{2}+1}\times \frac{t_{\text{d}}\cdot t_{\text{c}}}{t_{\text{d}}+t_{\text{i}}}$$

Equations (16) and (18) are valid only when the dosing time is in a valid range (ν'_MTR_ × *t*_d_ ≤ [CO_2_]*).

### 2.2. Experimental Results

A set of *D. salina* batch cultures were carried out to test the hypothesis that sufficient CO_2_ can be attained in a culture by “micro-bubbling” gas periodically (with little CO_2_ wasted and less energy cost) whilst similar algal growth can be achieved compared to when the gas is supplied continuously, but with higher CO_2_ capture efficiency.

**Figure 1:**
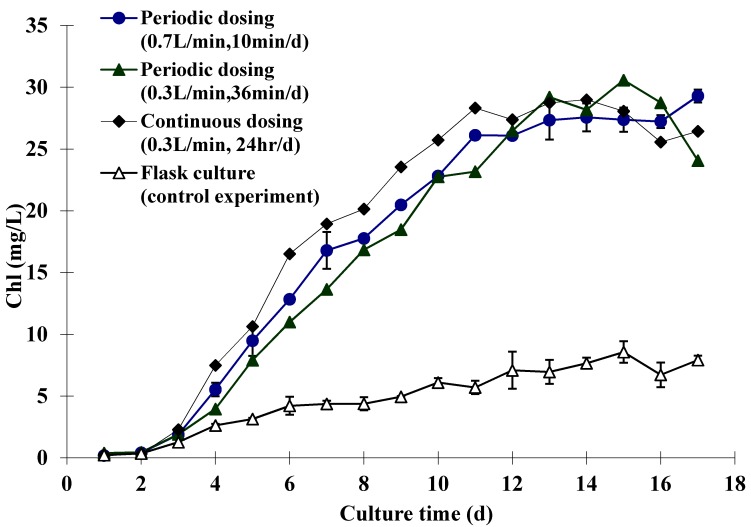
Daily chlorophyll content of the *D. salina* cultures with different dosing conditions. The cultures with 0.7 L·min^−1^ of periodic dosing were conducted in parallel, as were the control cultures. Therefore, the daily chlorophyll content for these two dosing conditions shown in this graph is the average value, with the error bars given separately.

Figure 1 shows the daily chlorophyll content of *D. salina* cultures supplied continuously and periodically with 5% CO_2_. As can be seen, the growth of these cultures, indicated by their chlorophyll content, appears to be fairly similar. This strongly supports the idea that *D. salina* growth is proportional to the total amount of CO_2_ that has been effectively transferred from gas phase into liquid phase, while extra CO_2_ dosing beyond the valid range does not improve the productivity. The final chlorophyll contents for the cultures with different dosing conditions were expected to be the same, as the total amount of CO_2_ mass transfer was kept identical. The contrast between the growth of the cultures fed with 5% CO_2_ and the control cultures can also be seen from Figure 1. Unsurprisingly the chlorophyll content of the control cultures remained much lower than other cultures, and a similar phenomenon was also observed in the study of Ying *et al.* [7].

Arguably the most important finding is the comparison that can be drawn between the growth of the *D. salina* cultures supplied continuously and daily with gas. It appears that there was no significant difference between the two types of culture. The daily chlorophyll content of continuously dosed culture seemed slightly higher between day 4 and day 11, which indicates the *D. salina* grew a bit faster under continuous dosing (approximately 1 day in advance of the periodic dosed cultures). However, the cultures engaged with periodic dosing model were still competitive to the continuously dosed culture, as they achieved a similar level of final chlorophyll content although with one day of delay. Regarding the CO_2_ capture efficiency, the cultures with different dosing conditions were compared (Table 1). As can be seen, by applying a periodic dosing strategy, CO_2_ capture efficiency achieved is about 10%–20%. It is expected that the capture efficiency could be further enhanced by improving the CO_2_ mass transfer (e.g., by further reducing the microbubble size). In contrast, with continuous dosing, capture efficiency was only 0.25% of CO_2_ supplied, which indicates that most of the CO_2_ was wasted rather than been captured. With a view to using microalgae for CO_2_ sequestration, this will mean not only wasted energy to dose CO_2_, but also any CO_2_ that was prevented from entering the atmosphere by fixation in the algae culture, will be greatly exceeded by the amount of CO_2_ passing straight through the culture into the atmosphere. Therefore, this result shows the potential for both economic and energy savings by adopting a periodic dosing strategy, as it appears that similar algal growth to a culture supplied continuously with CO_2_ can be achieved with periodic dosing, but with minimal CO_2_ waste and minimal energy consumption on dosing.

**Table 1:** Comparisons of CO_2_ capture efficiency for different dosing conditions.

Dosing Type (5% CO_2_)	Flow Rate (1 atm, 25 °C)	Dosing Time (in Total)	CO_2_ Total		
			Absorption	Input	η
Periodic	0.7 L·min^−1^	10 min·d^−1^ × 8 d	0.89 g	5.03 g	18%
Periodic	0.3 L·min^−1^	36 min·d^−1^ × 8 d	0.86 g	7.75 g	11%
Continuous	0.3 L·min^−1^	24 h·d^−1^ × 8 d	0.81 g	311.1 g	0.26%

NB: CO_2_ input_total_ = (CO_2_% × Q × *t*_d_ × *t*_c_ × P)/(R × T); CO_2_ absorption = ∆[CO_2_]_uptake_ Equation (16) × V_L_; η = CO_2_ absorption/CO_2_ input.

**Figure 2:**
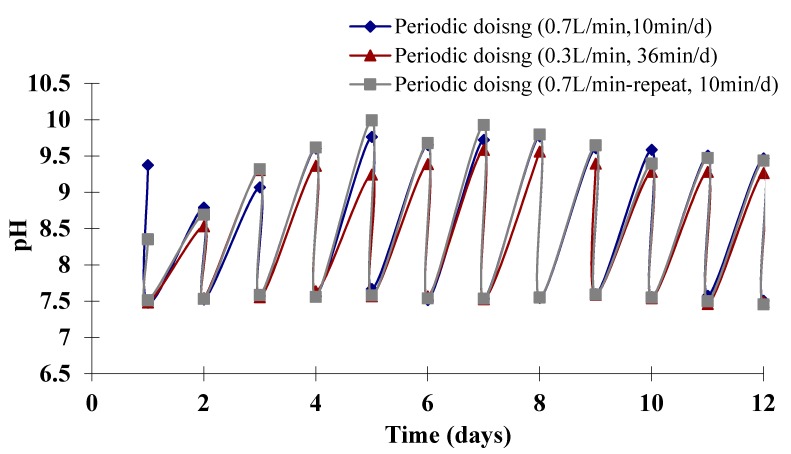
Daily pH values for *D. salina* cultures supplied periodically with 5% CO_2_. There are two pH values for each day, a higher one and a lower one, representing the pH values before and after CO_2_ dosing, respectively.

Additionally, the pH control achieved using periodic dosing was also observed during these experiments (Figure 2). The pH in the culture supplied periodically with gas was maintained in the target region of 7.5–9.5 without the use of expensive buffers. This also indicates the periodic dosing model for dosing time and dosing interval estimation are accurate, so that the pH was controlled in an expected range. These results agree with the previous studies by Ying *et al.* [7] who conducted a similar experiment culturing *D. salina* in Airlift Loop Bioreactors (ALBs) proved 30 min·d^−1^ of gas (5% CO_2_, 95% N_2_).

Finally, the predicted final concentrations of chlorophyll content for different periodic dosing conditions Equation (18) were compared with the experimental results, shown in Table 2. The errors between theoretical values and experimental values were about 2%–3%, which indicates the accuracy of Equation (18).

**Table 2:** Comparisons between estimated final concentrations of chlorophyll and real values for *D. salina* cultures with different dosing conditions.

Logarithmic Growth Time Period (d)	Dosing Condition	Estimated [Chl]_t_ (mg/L)	Real [Chl]_t_ (mg/L)	Error
8 d	0.7 L·min^−1^, 10 min·d^−1^	26.90	26.09	3%
8 d	0.3 L·min^−1^, 36 min·d^−1^	26.07	26.48	2%

## 3. Experimental Section

### 3.1. Experimental Setup

**Figure 3:**
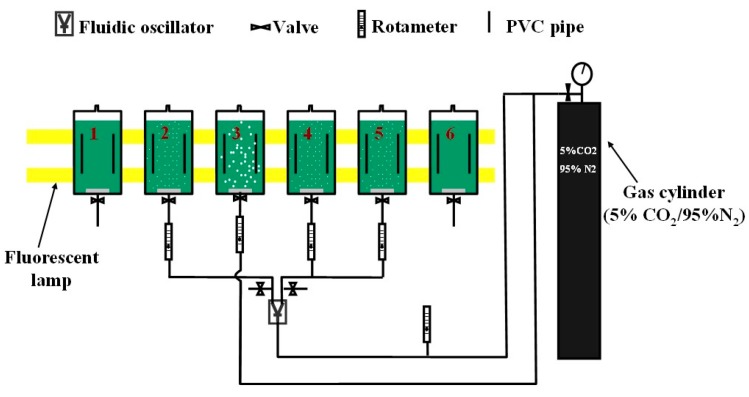
Setup for D. salina cultures. Six bioreactors containing *D. salina* cultures (50 mL of inocula to 2.5 L culture medium) were used for this experiment. Two of the six (No. 1 and 6) were flask cultures with no CO_2_ enriched gas being bubbled through the culture. Like the other four cultures, these control cultures were kept in identical ALBs to ensure that the illumination through these cultures was the same as those being supplied with gas. The remaining four reactors were supplied with CO_2_ enriched gas (5% CO_2_, 95% N_2_). Among them, No. 3 was dosed continuously with fine-bubbles, while No. 2, 4 and 5 were connected to a fluidic oscillator, dosed with microbubbles using periodic dosing strategy. No. 2 and 5 were conducted under same dosing condition for error analysis. The detailed dosing conditions for each reactor are listed in Table 4. The dosing time selected for each condition was estimated based on Equation (5). The detailed calculation is shown in Section 3.2. The temperature for each culture was maintained at ambient temperature around 24 °C. Two fluorescent lamps (90 μmol·m^−2^·s^−1^) were situated behind all reactors for illumination. Growth medium for *D. salina* cultures is shown in Table 3.

The experimental setup is illustrated in Figure 3. Samples of the *D. salina* were pre-cultured in shake flask (100 mL culture in 250 mL flasks) in a 25 ± 2 °C growth room. The growth medium composition is identical to the one described in Ying *et al.* [7], shown in Table 3. For the start of the main culture, 50 mL of this *D. salina* was added to 2.5 L of fresh culture medium in ALB. The dosing time (*t*_d_) and dosing interval (*t*_i_) were estimated based on Equations (5) and (15) and applied for the cultures engaged with periodic dosing strategy (No. 2, 4 and 5). pH was measured for each culture daily (for the culture dosed periodically, pH was measured twice per day, before and after dosing). 15 mL of sample for each culture was taken after gas dosing, followed by topping up the culture with 15 mL of fresh medium (For flask cultures, a sterilized glass stick was inserted into the culture for a proper stirring. Samples were taken after that.). The chlorophyll content for each sample was determined by measuring each sample’s optical density for wavelengths of 645 and 663 nm using the same method to that used in Zimmerman *et al.* [16] and Ying *et al.* [7].

**Table 3:** *D. salina* culture medium.

Composition of Growth Medium Per Litre
0.5 M·NaCl; 0.185 mM·H_3_BO_3_; 10 mM·KCl; 0.007 mM MnSO_4_; 20 mM·MgCl_2_; 0.8 × 10^−3^ mM·ZnCl_2_; 10 mM CaCl_2_; 0.2 × 10^−4^ mM CoCl_2_; 24 mM MgSO_4_; 0.2 × 10^−6^ mM·CuCl_2_; 5 mM NaNO_3_; 24 mM·Na_2_SO_4_; 0.0119 mM·NaHCO_3_; 0.1 mM NaH_2_PO_4_; 0.0015 mM·FeEDTA

**Table 4:** The dosing conditions for each culture. The average size for the microbubbles and fine bubbles were measured by using high speed camera [17].

Reactor	Dosing Conditions				Represent
	Bubbles	Fluidic Oscillator	Dosing Flowrate	Dosing Time Equation (5)	
No. 1	No bubbles	Not Engaged	0	0	Flask culture
No. 2	Microbubble (d_32_: 388 μm)	Engaged	0.7 L·min^−1^	10 min·d^−1^	Periodic dosing
No. 3	Fine-bubble (d_32_: 719 μm)	Not Engaged	0.3 L·min^−1^	24 h·d^−1^	Continuous dosing
No. 4	Microbubble (d_32_: 388 μm)	Engaged	0.3 L·min^−1^	36 min·d^−1^	Periodic dosing
No. 5	Microbubble (d_32_: 388 μm)	Engaged	0.7 L·min^−1^	10 min·d^−1^	Duplication of No. 2
No. 6	No bubbles	Not Engaged	0	0	Duplication of No. 1

### 3.2. Estimation of the Dosing Time and Dosing Interval

In Table 4, the dosing time for 0.3 and 0.7 L·min^−1^ were calculated based on Equation (5). The detailed calculation was shown as follows.

Since the selected pH range for *D. S* culture was 7.5–9.5, and the concentration of NaHCO_3_ in the culture medium was 0.0119 mol·L^−1^, [*C*_T_] at pH = 7.5 and pH = 9.5 can be calculated based on Equation (7), which gives: [*C*_T_]_pH = 7.5_ = 0.0128 mol·L^−1^; [*C*_T_]_pH = 9.5_ = 0.0106 mol·L^−1^.

The K_L_a for CO_2_ mass transfer in the real algal culture was previously determined to be 0.044 min^−1^ for 0.3 L·min^−1^ of bubbling flow rate and 0.174 min^−1^ for 0.7 L·min^−1^ [9]. Finally, t_d_ was calculated to be about 36 min for 0.3 L·min^−1^ of bubbling flow rate and 10 min for 0.7 L·min^−1^.

For dosing interval estimation, it can be calculated by Equation (15). 

Based on the assumption that Chl_0_ = 5 mg·L^−1^ and *t*_c_ = 8 days, *t*_i_ is calculated to be 1.5 days. Conservatively, 1 day of dosing interval was used in the experiment so that DO can be removed by CO_2_ dosing in time.

## 4. Conclusions

A periodic CO_2_ dosing strategy for *D. salina* culture is proposed, with a model of periodic CO_2_ dosing including dosing time calculation, dosing interval estimation and final chlorophyll yield prediction established. The cultures applying periodic CO_2_ dosing strategy provide a similar productivity to the culture with continuous dosing, but with a greater CO_2_ sequestration efficiency. Due to the time limitation, only two different gas dosing flow rates were tested in this study. More flow rates can be tested to explore the relation between dosing flow rate and CO_2_ capture efficiency in the future studies.

## Abbreviations

Δ[Chl]: changes in the concentration of chlorophyll content, mg·L^−1^
Δ[CO_2_]_dosed_: the difference between the concentration of dissolved CO_2_ before and after dosing, mol·L^−1^
Δ[CO_2_]_uptake_: changes in the concentration of CO_2_(aq) due to the CO_2_ consumption by algal growth, mol·L^−1^
Δ[Na^+^]: the concentration of sodium ions in the liquid which comes from NaHCO_3_, mol·L^−1^
CO_2_%: the concentration of CO_2_ in the gas stream, % (*v*/*v*)
d_32_: the Sauter mean diameter of the bubbles, μm
η: the CO_2_ capture efficiency, %
K_L_a: volumetric mass transfer coefficient (min^−1^), where “K_L_” is the CO_2_ gas-liquid mass transfer coefficient (m·min^−1^); “a” means the gas-liquid interfacial area (m^−1^)
P: standard atmospheric pressure, 101.325 Kpa
pH: the pH value of the liquid
R: universal gas constant, 8.314 J·mol^−1^·K^−1^
T: the temperature, K
*t*_c_: culture time period, d
*t*_d_: CO_2_ dosing time, min
*t*_i_: CO_2_ dosing interval, min
ν'_Chl_: average productivity of chlorophyll content, mg·L^−1^·d^−1^
ν_CO2 uptake_: instantaneous algal CO_2_ uptake rate, mol·L^−1^·min^−1^
ν'_CO2 uptake_: average algal CO_2_ uptake rate, mol·L^−1^·min^−1^
Q: the gas dosing flowrate, L·min^−1^
ν'_MTR_: CO_2_ average mass transfer rate, mol·L^−1^·min^−1^
V_L_: the volume of the liquid, L
[*C*_T_]: total concentration of inorganic carbon in the liquid, mol·L^−1^
[*C*_T_]_pH=A_: total concentration of inorganic carbon in the liquid at pH=A, mol·L^−1^
[*C*_T_]_pH=B_: total concentration of inorganic carbon in the liquid at pH=B, mol·L^−1^
[Chl]: concentration of chlorophyll content in the culture, mg·L^−1^
[Chl]_0_: initial concentration of chlorophyll content at the beginning of log growth phase, mg·L^−1^
[CO_2_]: dissolved carbon dioxide concentration in the liquid, mol·L^−1^
[CO_2_]^*^: dissolved carbon dioxide equilibrium concentration in the liquid, mol·L^−1^
[CO_2_]_0_: initial dissolved carbon dioxide concentration in the liquid, mol·L^−1^
[CO_2_]_pH=A_: dissolved carbon dioxide concentration in the liquid at pH=A, mol·L^−1^
[CO_2_]_pH=B_: dissolved carbon dioxide concentration in the liquid at pH=B, mol·L^−1^
[CO_3_^2−^]: the concentration of carbonate, mol·L^−1^
[HCO_3_^−^]: the concentration of bicarbonate, mol·L^−1^
